# A method for evaluating cocaine-induced social preference in rats

**DOI:** 10.14440/jbm.2017.145

**Published:** 2017-03-07

**Authors:** Paige M. Dingess, Morgan J. Deters, Rebecca A. Darling, Erin A. Yarborough, Travis E. Brown

**Affiliations:** 1 University of Wyoming, Neuroscience Program, Laramie, WY 82071, USA; 2 University of Wyoming, School of Pharmacy, Laramie, WY 82071, USA

**Keywords:** social cues, conditioned preference, cocaine

## Abstract

Drug addicts are extremely sensitive to cues that predict drug availability and exposure to these cues can facilitate drug relapse. Cues vary in their nature but can include drug-associated paraphernalia, environmental contexts, and discrete conditioned stimuli (*e.g.*, advertisements). One cue that has recently been heavily investigated is that of social interaction. To date, it has been demonstrated that when cocaine is conditioned with social interaction, place preference for cocaine significantly increases, suggesting that the presence of social interaction during a drug-associated “high” enhances the magnitude of drug reward. When social interaction is provided in a mutually exclusive, non-drug environment though, it can serve as a preventative stimulus towards cocaine seeking. What remains unknown is whether contact with rats associated with drug experience facilitates preferential social interactions for those rats. The first step in answering this question is to determine if rats can behaviorally discriminate between drug-associated and non-drug-associated conspecifics, much like humans can differentiate their “drug-friends” from their non-drug-using friends. Using a custom social interaction chamber, in which rats were able to interact with two distinct conspecifics *via* holes in a boundary wall, we demonstrate that rats exhibit more interactive and investigative behavior towards a partner that was consistently present during the drug-state, than a partner that was present when the rat was “sober”. It is our hope that this protocol will contribute to the development of models designed to study social cue-induced reinstatement, and related neural substrates, and will ultimately contribute to the treatment of substance use disorders.

## INTRODUCTION

One of the primary challenges in treating addiction is the development of intense cravings during abstinence that override the ability of a patient to abstain from their drug of choice [1]. This phenomenon often precedes drug-seeking behavior and ultimately facilitates relapse [2,3]. As a result, many addiction therapies are targeted towards managing these cravings, typically by advocating the avoidance of cues that trigger them [4,5]. Preclinical rodent studies repeatedly demonstrate that exposure to stimuli previously paired with drugs of abuse, such as a light and tone predictive of drug availability, has the power to promote drug-seeking behavior [6-8]. This observation mimics clinical studies, which indicate that exposure to drug-related cues, including video scenes of drug paraphernalia or subjects actively self-administering drugs, enhances subjective craving in humans [9,10]. These types of cues have certainly been shown to facilitate relapse behavior [11], but there exist other cues that require exploration. Research is beginning to explore and understand the role that social cues have on drug-seeking behavior. For example, there is evidence to support that having 1 or more friend that currently uses, or even previously used, cocaine hinders abstinence among Caucasian cocaine addicts in recovery [12] and it has been suggested, though not conclusively studied, that breaking ties from drug-using friends is a difficult and necessary task of addiction recovery [13]. Recently, Fatseas *et al*. in 2015 demonstrated that interaction with friends previously associated with drug use amplifies subjective cravings for several drugs of abuse including alcohol, tobacco, cannabis, and heroin in humans [14].

Rodents are an ideal animal model to study social interaction as they have been characterized as highly social animals that exhibit easily measured and analyzed behaviors [15-18]. Importantly, studies have demonstrated that social interaction is a powerfully salient stimulus, particularly for singly housed rats during adolescence, a period in which social reward is very robust [19]. This is evidenced by findings that singly housed adolescent rats will readily prefer to spend time in social-paired environments [20,21], even when contact is restricted by a mesh wall [22], and given the choice between an amphetamine-paired context and a social-paired context, will spend more time in the latter [21]. In the choice between a cocaine- or social-paired context, it has been demonstrated that adult, group-housed, rats spend equal amounts of time in each of those environments, suggesting that social reward is strong enough to at least challenge the extremely euphoric effects of cocaine even in the adult state and even when animals have access to social interaction in the home cage [23]. In addition to social interaction serving as a reward on its own, it has been repeatedly demonstrated that when conditioned with social interaction, place preference for cocaine is significantly increased, whether animals are singly-housed [20] and even when they are grouped [24]. While the presence of social interaction during a drug-associated “high” appears to enhance the magnitude of drug reward, when it is provided in a mutually exclusive and drug-free setting, it has been shown to reverse place preference from cocaine to social interaction and prevent the reacquisition of cocaine conditioned place preference (CPP) [25,26]. Thus, the effect that social interaction has on drug reward has much to do with the context (drug-associated or not) from which the rat affiliates it.

These studies together highlight the complex relationship between social and drug reward but leave the preclinical field with an unanswered question—can drug-paired social cues facilitate drug craving, seeking, and/or reinstatement in rodents as they have been shown to do in the human experience of addiction [14]? An important step in addressing this question is to determine whether rats can distinguish between drug-paired and non-drug-paired conspecifics, the answer to which provides insight on the feasibility of developing a model for social cue-induced reinstatement.

To address this, we implemented a modified conditioned preference paradigm in which rats learned to associate one animal with cocaine (“cocaine buddy”) and another with saline (“control buddy”). Using a custom social interaction chamber that allowed rats to interact *via* holes in a partition wall, we found that rats engaged in significantly more investigative and interactive behaviors towards their cocaine buddy compared to their control buddy, in an exposure-dependent fashion. This finding allows the field to move forward in developing a model of social cue-induced drug seeking, which may ultimately contribute to the investigation of neural correlates of this specific type of cue-triggered drug-seeking and to the treatment of substance use disorders.

## MATERIALS AND METHODS

### Animals

Experiments were performed with adult male Sprague-Dawley rats, postnatal day 60 at the beginning of experimentation (body weight range: 350–400 g). Female rats were excluded from this study due to a previous finding that concurrent conditioning with social interaction and cocaine enhances place preference more robustly in males [24], but will be examined in future studies. Each experimental rat was paired with a control buddy and a cocaine buddy for 10 pairings in Experiments 1 (*n* = 4) and 2 (*n* = 7) or 20 pairings in Experiment 3 (*n* = 16). During these pairings, test rats were injected with cocaine (12 mg/kg) or saline (1 ml/kg) and placed into a conditioning chamber (described below) with their cocaine or control buddy, respectively.

All rats were housed in groups of 4 in a temperature-controlled room under a 12 h light/dark cycle such that test rats were housed together, control buddies were housed together, and drug buddies were housed together. This housing design, combined with the fact that buddies came from distinct litters than the test rat, was implemented in order to minimize social bias. Rats were group housed in an effort to avoid the effect that social isolation might have on the salience of social reward [21]. Rats had access to food and water at all times. All procedures were performed in accordance with the NIH Guide for the Care and Use of Laboratory Animals and with approval from the Institutional Animal Care and Use Committee (IACUC) at the University of Wyoming.

### Pairing procedures

**Figure 1.**
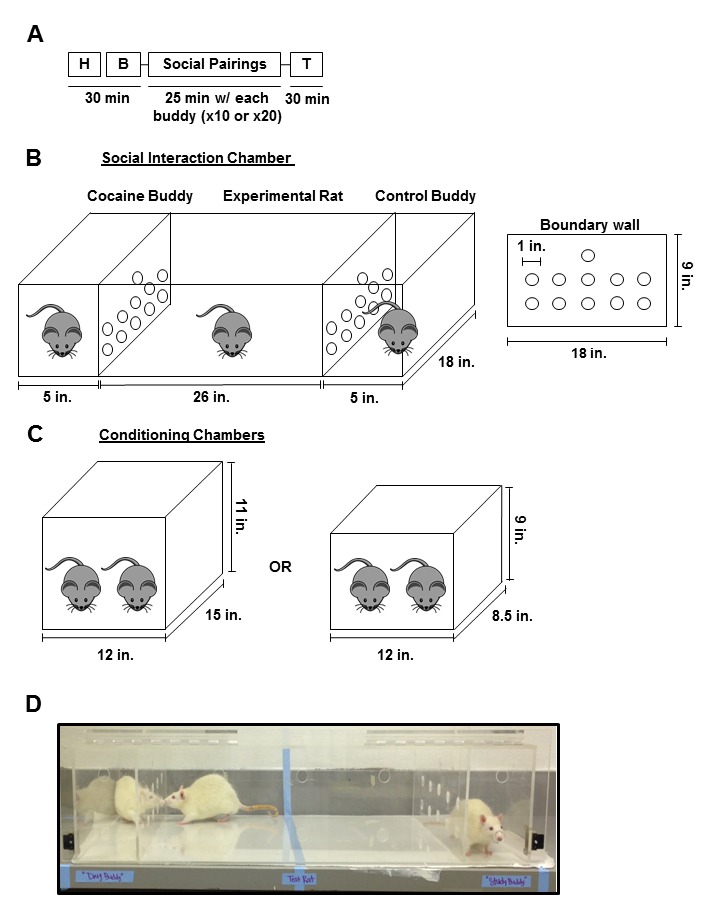
Experimental design and apparatus. **A.** Experimental timeline. Rats were placed into the central compartment of the social interaction chamber for 30 min during habituation (H) and baseline (B) days, with access to both buddies. Rats were then conditioned by pairing them with the control buddy or cocaine buddy for 25 min after receiving an *i.p.* injection saline (1 ml/kg) or cocaine (12 mg/kg), respectively. This was conducted in a separate chamber. On test (T) day, rats were again placed into the central compartment of the social interaction chamber, with access to both buddies, and interaction behavior was measured. **B.** Illustration of the social interaction chamber (left) and boundary walls (right), which separated the test rats from buddy rats. These walls were equipped with 1-inch perforations to allow for interaction. **C.** Illustration of the conditioning chambers used. **D.** Photograph of social interaction chamber, including an example of a face-to-face interaction between the test rat and drug buddy.

We used a modified CPP procedure to examine whether rats would exhibit a conditioned social preference for conspecifics paired with cocaine over those paired with saline (**Fig. 1A**). Specifically, we utilized a custom-made clear Plexiglas 3-chambered social interaction box that allowed for the test rat to interact, *via* eleven 1-inch holes in a boundary wall, with two distinct rats in separate chambers (buddy chambers: 5" × 18" × 9"; central test rat chamber: 26" × 18" × 9", **Fig. 1B**). On the first day of training, experimental rats were placed into the central compartment of the social interaction chamber for 30 min in a drug-free state (habituation). During this time, rats were able to explore the chamber as well as interact with the two buddy rats, both of different litters and housed separately from the test rat. At this point, neither of the buddy rats were conditioned to have any significance (*i.e.*, the rats were strangers to one another). On the following day (baseline), rats were placed into the central chamber in the same manner as above, with the same two buddies, in the same location, from the day before. During this time, several interaction behaviors (defined below) were video recorded and manually scored. The placement of drug and control buddies was counterbalanced such that drug buddies were located in the left chamber for some test rats and in the right for others.

“Nose pokes” were used as a measure of social investigation, with importantly, the possibility of reciprocated interaction. A nose poke was operationally defined as an event in which the nose of the test rat physically crossed the threshold of the central compartment to the control or cocaine buddy compartment *via* the 1-inch openings in the boundary wall. “Pawing” was defined as an event in which the test rat placed both of its paws on the boundary wall to the control or cocaine buddy compartment. Total “exploration time” was also used as a measure of investigation and was scored in seconds as any active exploratory behavior towards the boundary wall, including nose poking, pawing, sniffing, and walking or rearing along or towards the boundary wall. “Initiation events” were used as a measure of reciprocated interaction (face-to-face contact). A rat was defined as the initiator if it placed its nose in the 1-inch opening first and awaited contact by another rat (**Fig. 1D**).

Experiment 1 followed an unbiased design such that the assigned cocaine buddy was the initially preferred rat in some cases and the initially non-preferred rat in others. The preference for one buddy over the other was determined by the amount of nose poking (*via* the 1-inch holes) towards each buddy during baseline. Experiments 2 and 3 followed a biased design such that the initially non-preferred rat was chosen as the cocaine buddy and the preferred rat as the control buddy. The selection of a biased design was the result of a comparison of results in Experiments 1 and 2.

Rats were then paired with each of these buddies on alternating days for 20 consecutive days (10 pairings with each buddy, Experiments 1 and 2) or 40 consecutive days (20 pairings with each buddy, Experiment 3). Other studies have examined conditioned social preference, measured by time spent in a social-paired context [20,21,24], and revealed that the magnitude of social preference increases with number of pairings (up to 8 pairings) [20]. The behavior measured in the present study is slightly distinct, and thus an initial pairing number of 10 was selected (and increased to 20 after results obtained from Experiments 1 and 2) to ensure that social preference was acquired. During these pairings, the experimental rat was injected (intraperitoneally, *i.p.*) with either cocaine (12 mg/kg) or saline (1 ml/kg) and immediately placed into a chamber (**Fig. 1C**) with the cocaine buddy or control buddy, respectively, for 25 min. Neither the control nor cocaine buddy was ever injected with saline or cocaine (*i.e.*, they were always in a drug-free state).

The chambers used for pairing procedures were either white modular Med-Associates (St. Albans, VT) chambers (12 × 8.5 × 9 in, surface area = 102 in^2^) or opaque plastic containers (12 × 15 × 11 in, surface area = 180 in^2^). The size of these chambers was selectively chosen based upon a previous report that a conditioning space of 116.25 in^2^ produces robust place preference for social interaction but that conditioning in a confined space (~58.13 in^2^), despite allowing for more direct contact, fails to elicit the same response [27]. All experiments used the 102 in^2^ Med-Associates chambers (**Fig 1C**, right) as conditioning chambers, with the exception of a cohort in Experiment 3, which used the opaque plastic containers (**Fig 1C**, left). Despite the difference in surface area, the use of this chamber did not have an effect on experimental outcomes. These pairing chambers were distinct from the social interaction chamber and were not equipped with a boundary wall, which allowed the rats to freely engage one another as they would in a home cage environment (**Fig. 1C**). These chambers were purposefully open field due to the observation that the most salient sensory stimulus in inducing CPP for social interaction is taction (touch) [27].

Following conditioning, rats were placed back into the central compartment of the social interaction chamber in the same manner as habituation and baseline, in a drug-free state, with the now conditioned drug and control buddies located in the same compartments that they were in during the first two sessions in this chamber. During this time (test), social interactions (nose poking, pawing, total exploration time, and initiation of face-to-face contact) were again video recorded and manually scored.

### Statistical analyses

The results are expressed as mean ± SEM. GraphPad Prism was utilized for all statistical analysis. 2-way repeated measures ANOVA was used for all experiments, except the preference scores, which were analyzed with a paired *t* test. Significant interactions (*P* < 0.05) were further analyzed using Sidak’s multiple comparisons test.

## RESULTS

**Figure 2.**
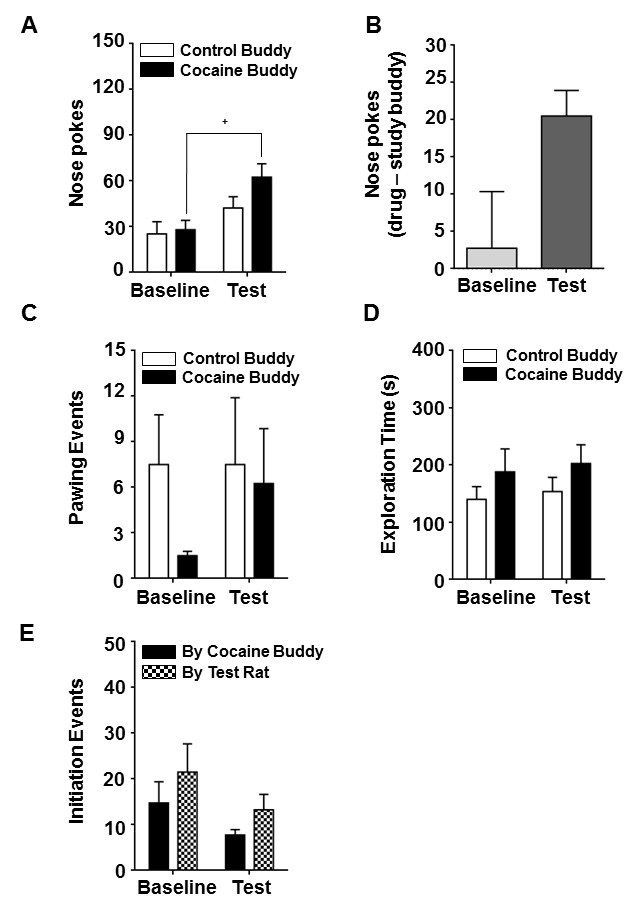
Experiment 1: Interaction behavior following 10 pairings with an unbiased design. **A.** Nose pokes towards control buddy (white) and cocaine buddy (black) during baseline and test days. **B.** Preference score, measured by subtracting the amount of nose pokes toward the control buddy from nose pokes toward the cocaine buddy during baseline (light gray) and test (dark gray). **C.** Pawing events against the boundary wall to the control buddy and cocaine buddy during baseline and test. **D.** Exploration time of the boundary wall to the control buddy and cocaine buddy during baseline and test. **E.** Initiation of reciprocated contact from the cocaine buddy to the test rat (black) and the test rat to the cocaine buddy (checkered) during baseline and test. (^+^*P* < 0.05: significant increase in interaction toward cocaine buddy from baseline to test).

In Experiment 1, rats were conditioned with their buddies, for 10 parings each using an unbiased design, and were subsequently assessed for interaction behavior towards each buddy in the social interaction chamber (test). The purpose of Experiment 1 was to determine whether 10 pairings was sufficient to induce an increase in interaction behavior towards the cocaine buddy. 2-way repeated measures ANOVA revealed that there was a significant day effect (F_(1, 6)_ = 11.91, *P* < 0.05) for nose poking. Specifically, rats demonstrated greater nose poking towards the cocaine buddy during the test than they did during baseline (baseline: 28.00 ± 12.33; test: 62.50 ± 17.54, **Fig. 2A**). Despite this conditioning-dependent increase, the preference to nose poke towards the control buddy or towards the cocaine buddy during the test was not significantly different, suggesting that the pairing procedure did not adequately facilitate the behavioral discrimination between drug-paired and saline-paired conspecifics. This is reflected in the lack of change in the preference score, (calculated by subtracting the nose pokes toward the control buddy from the nose pokes toward the cocaine buddy during baseline and test, **Fig. 2B**) and further evidenced by the lack of significant change in any other parameters measured (**Fig. 2C-2E**).

**Figure 3.**
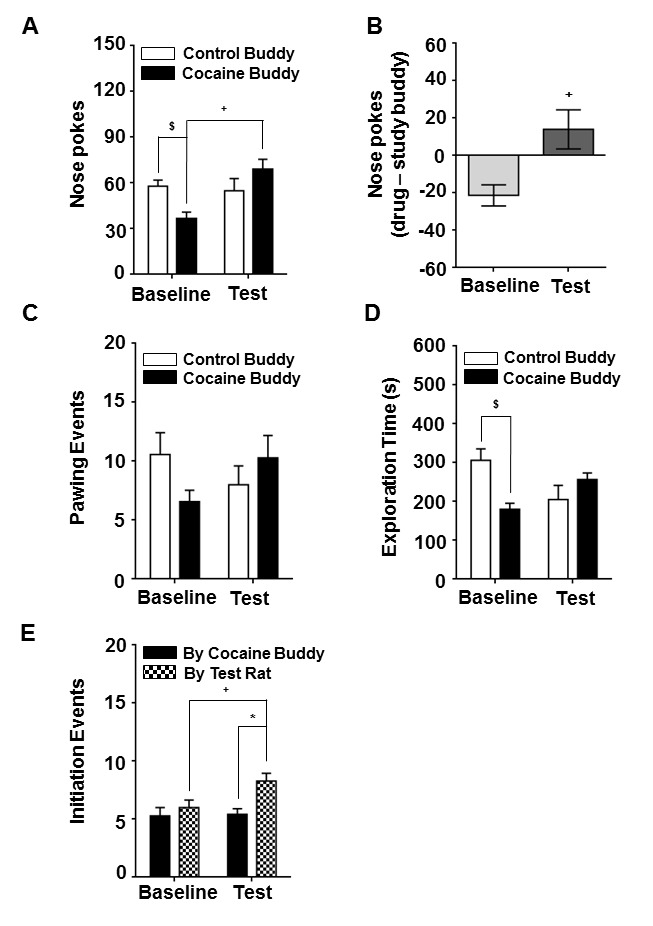
Experiment 2: Interaction behavior following 10 pairings with a biased design. **A.** Nose pokes towards control buddy (white) and cocaine buddy (black) during baseline and test days. **B.** Preference score, measured by subtracting the amount of nose pokes toward the control buddy from nose pokes toward the cocaine buddy during baseline (light gray) and test (dark gray). **C.** Pawing events against the boundary wall to the control buddy and drug buddy during baseline and test. **D.** Exploration time of the boundary wall to the control buddy and cocaine buddy during baseline and test. **E.** Initiation of reciprocated contact from the cocaine buddy to the test rat (black) and the test rat to the cocaine buddy (checkered) during baseline and test. (^$^*P* < 0.05: significant difference in interaction between control and cocaine buddies during baseline; *P < 0.05: significant difference in interaction between control and cocaine buddies during test; ^+^*P* < 0.05: significant increase in interaction toward cocaine buddy from baseline to test).

Based upon our results from Experiment 1, we chose to explore the option of a biased design to determine if pairing parameters impacted behavioral outcome. Post-hoc analysis revealed a significant day effect (F_(1, 12)_ = 7.57, *P* < 0.05) as well as a significant buddy × day interaction (F_(1, 12)_ = 10.98, *P* < 0.01) for nose poking. As a product of this experimental design, rats nose poked to their control buddies significantly more than to their cocaine buddies during baseline (control buddy: 57.86 ± 4.12; cocaine buddy: 36.57 ± 4.27, **Fig. 3A**), which was no surprise given that nose poking was the parameter used to assign buddies. This facilitated an increase in the preference score (baseline: −21.29 ± 5.66; test: 14.00 ± 10.45, *P* < 0.05, **Fig. 3B**), which was deigned to compare the relative preference between the two buddies during both baseline and test. Rats also spent more time exploring the boundary wall of the control buddy during baseline (control buddy: 306.14 ± 29.80; cocaine buddy: 180.29 ± 15.44, **Fig. 3D**), likely again a product of the experimental design. Similar to the results from Experiment 1 though, we observed an increase in nose poking towards the cocaine buddy from baseline to test (baseline: 36.57 ± 4.27; test: 68.86 ± 6.71, **Fig. 3A**) but no significant differences in the preference to nose poke, paw, or explore the boundary wall of either buddy during test, indicating at this point that these are not appropriate measures to assess social preference or that 10 pairings is an insufficient amount to produce reliable discrimination between buddies. Despite these negative results, interestingly, changes in initiation events began to emerge. There was a significant day effect (F_(1, 12)_ = 7.67, *P* < 0.05), a significant buddy effect (F_(1, 12)_ = 5.11, *P* < 0.05), and a significant buddy × day interaction (F_(1, 12)_ = 5.97, *P* < 0.05). Specifically, initiation events by the test rat, to the cocaine buddy, increased from baseline to test (baseline: 6.00 ± 0.66; test: 8.29 ± 0.68, **Fig. 3E**), indicating a heightened motivation to seek out interaction from the newly conditioned rat. As a result of this time-dependent increase, we also observed a significant difference in the number of initiation events produced by the test rat *vs*. those produced by the cocaine rat during the test (by test rat: 8.29 ± 0.68; by cocaine buddy: 5.43 ± 0.48, **Fig. 3E**). Results from this experiment were more promising than those obtained from Experiment 1 but did not provide strong, convincing evidence that the test rat could discriminate between the two buddies. We therefore chose to increase the number of pairings from 10 to 20 using a biased design.

**Figure 4.**
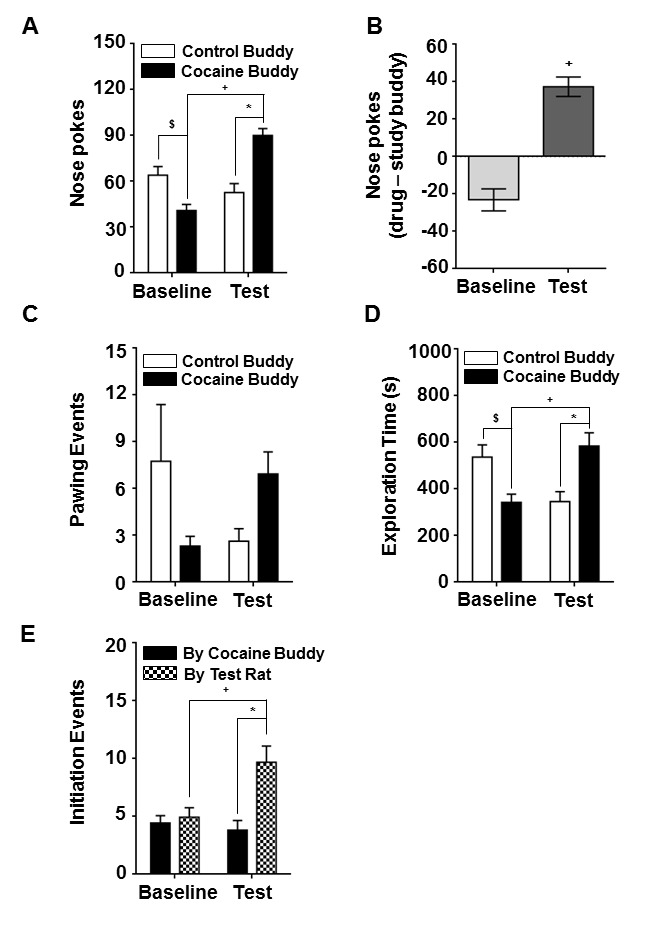
Experiment 3: Interaction behavior following 20 pairings with a biased design. **A.** Nose pokes towards control buddy (white) and cocaine buddy (black) during baseline and test days. **B.** Preference score, measured by subtracting the amount of nose pokes toward the control buddy from nose pokes toward the cocaine buddy during baseline (light gray) and test (dark gray). **C.** Pawing events against the boundary wall to the control buddy and drug buddy during baseline and test. **D.** Exploration time of the boundary wall to the control buddy and cocaine buddy during baseline and test. **E.** Initiation of reciprocated contact from the cocaine buddy to the test rat (black) and the test rat to the cocaine buddy (checkered) during baseline and test. (^$^*P* < 0.05: significant difference in interaction between control and cocaine buddies during baseline; *P < 0.05: significant difference in interaction between control and cocaine buddies during test; ^+^*P* < 0.05: significant increase in interaction toward cocaine buddy from baseline to test).

The parameters of Experiment 3 were identical to Experiment 2 except that rats were conditioned with their buddies for 20 parings each. 2-way repeated measures ANOVA showed that there was a significant day effect (F_(1, 30)_ = 20.17, *P* < 0.001) and a significant buddy × day interaction (F_(1, 30)_ = 58.81, *P* < 0.001) for nose poking. Post-hoc analysis revealed rats increased their nose poking towards their cocaine buddy from baseline to test day (baseline: 40.88 ± 16.10; test: 90.00 ± 18.23, **Fig. 4A**) but importantly, nose poked to their cocaine buddy significantly more than their control buddy on the test day (control buddy: 52.63 ± 23.70; cocaine buddy: 90.00 ± 18.23, **Fig. 4A**), which served as the first indication that following conditioning, rats could discriminate between the two buddies. Again, the relatively fewer nose pokes towards the cocaine buddy during baseline (control buddy: 64.00 ± 22.85; cocaine buddy: 40.88 ± 16.10) is attributed to the fact that a biased design was implemented and the cocaine buddy was chosen as the initially non-preferred rat. This experimental design yielded a more robust change in preference score (baseline: −23.13 ± 5.90; test: 37.38 ± 5.20, *P* < 0.001, **Fig. 4B**), indicating increased preference for the drug buddy over time. Similar to nose poking, there was a significant buddy × day interaction (F_(1, 30)_ = 37.50, *P* < 0.001) for exploration time. Post-hoc analysis revealed that test rats spent more time exploring the cocaine buddy boundary wall during the test than they did during baseline (baseline: 342.50 ± 143.00; test: 584.90 ± 222.90, **Fig. 4D**) and importantly, time spent exploring the cocaine buddy boundary wall was significantly greater than time spent exploring the control buddy boundary wall during the test (control buddy: 345.90 ± 171.90; cocaine buddy: 584.90 ± 222.90, **Fig. 4D**), further supporting a learned discrimination between the control and cocaine buddies and preference for the latter.

Lastly, an analysis of the initiation for reciprocated face-to-face contact was measured. There was a significant day effect (F_(1, 30)_ = 4.93, *P* < 0.05), a significant buddy effect (F_(1, 30)_ = 9.99, *P* < 0.01), and a significant buddy × day interaction (F_(1, 30)_ = 8.37, *P* < 0.001). Specifically, the initiation of interaction by the test rat towards the cocaine buddy increased from baseline to test (baseline: 4.94 ± 3.30; test: 9.69 ± 5.6, **Fig. 4E**). Additionally, the test rat initiated contact towards the cocaine buddy significantly more than the cocaine buddy initiated contact towards the test rat during the test day (initiation by the test rat: 9.69 ± 5.61; initiation by the cocaine buddy: 3.81 ± 3.37, **Fig. 4E**) suggesting that the test rat was more eager to engage in social interaction than the cocaine buddy. This is likely due to the fact that the test rat had no conditioned significance to the cocaine buddy.

## DISCUSSION

The goal of this study was to determine whether rats would display a greater preference for a conspecific learned to be associated with a drug (cocaine) experience, over one learned to be associated with a non-drug (saline) experience. The purpose of which was to provide relevant foundational information to facilitate the development of an animal model to investigate drug-paired social interactions, and their neural substrates, as a contributing factor in drug reinstatement. Rodent models of addiction already clearly demonstrate that drug-associated stimuli, including contextual cues, the drug itself (drug prime) and even stress, have incredible power in facilitating drug craving and drug reinstatement [6,28-33]. Thus far, studies exploring drug-paired social interaction have concluded that the presence of social interaction during the drug-state has an additive effect on drug reward [20,24] but whether reintroduction to drug-paired social cues promotes drug reinstatement remains to be elucidated. Although our results do not address this question directly, they do suggest that rats display a behavioral preference for drug-paired conspecifics and therefore provide promise that this type of cue could be investigated in rodent models of drug relapse.

Due to the unique nature of the testing procedure, in that preference was assessed by behavioral interactions and not by time spent in a chamber [20,21,24,34], it was unclear how many pairings would be required to induce social preference. Thiel *et al*., in 2008 demonstrated that when preference is assessed by time spent in a social-paired chamber, magnitude of preference increases with the number of pairings (up to 8 pairings) [20]. In order to ensure that social preference would be acquired in our model, we first attempted the use of 10 pairings (Experiment 1, **Fig. 2**), using an unbiased design. This procedure yielded only a mild increase in nose-poking behavior toward the drug buddy from baseline to test (**Fig. 2A**). Although this suggested that conditioning is sufficient to increase interest in the cocaine buddy, it did not convince us that the test rats preferred the cocaine buddy to the control buddy. We therefore altered our pairing procedure to a biased design (Experiment 3, **Fig. 3**). This produced slightly more promising results, notably an increase in initiative events by the test rat, to the cocaine buddy, over time (**Fig. 3E**), but was still insufficient in producing differential responding toward control *vs*. cocaine buddies during the test in other measures (**Fig. 3A**, **3C** and **3D**). Finally, we increased our pairing procedure to include 20 pairings (Experiment 3, **Fig. 4**) to determine if social preference is exposure-dependent. Indeed, this pairing procedure yielded not only and increase in investigative behaviors towards cocaine buddies from baseline to test but also measurable differences between behaviors aimed at cocaine *vs*. control buddies. (**Fig. 4A** and **4D**). These observations suggest that this conditioning paradigm enhances the motivation to seek social interaction with drug-associated conspecifics over time but additionally increases the relative preference for those conspecifics.

From these results, a few recommendations can be made. First, the ideal number of pairings to induce this level of preference and discrimination is at most 20, and likely the true minimum lies somewhere between 10 and 20. Future studies should be aimed at more precisely determining the optimal number of pairings. Second, the use of an unbiased design appears to more clearly illustrate the magnitude of social preference. Although this is an artificial manipulation in the experimental design, our results in Experiment 1, which show no change in nose poking towards the control buddy across time, indicate that the observed effects are truly conditioning-dependent rather than a product of design choice. Third, the behaviors measured in this study were selected based upon the possible behavioral outputs in this specific social interaction chamber. Our results suggest that nose poking is perhaps the most sensitive measure, evidenced by the fact that (1) increased nose poking towards the cocaine buddy from baseline to test was the first difference to emerge, even in the context of a biased design (**Fig. 2A**); and (2) it increased in magnitude with more pairings (**Fig. 4A**). Thus, when employing an experimental design such as the one presented, nose poking might be a good overall measure of social preference. Pawing was measured as another potential investigative behavior but on its own did not produce significant results under any of the experimental procedures. It is hypothesized that this may be due to a lack of physical contact with the buddies and therefore that social preference is best displayed in actions of actual (or attempted) contact. This hypothesis is supported by evidence suggesting that the most salient cue in inducing conditioned social preference is touch [34].

The subsequent step in the development of a model to study the role of social relationships as a cue for drug-seeking behavior is to apply a protocol, as described here, in a CPP or self-administration paradigm. One way to do this would be to condition rats in the same manner as defined above, and then subject them to a cocaine CPP or self-administration protocol, similar to those previously described [35-37]. Following an extinction period, reintroduction to the cocaine-paired buddy could then be used to evaluate reinstatement behavior. There are several important factors to consider when making this transition. First, although not directly measured in the present study, it is known that cocaine reduces fight attacks and aggressive behavior [38] in rodents. The effect that this has on subsequent social preference is unclear but our results suggest that any reduction in play that does arise as a result of cocaine is at least insufficient in reducing preference, given that it develops for drug-paired rats anyways. Secondly, in the human experience of drug addiction, it is unlikely that a drug-associated friend will be a sober, non-drug-using individual like the cocaine buddies used in this study. Thus, future studies should consider the use of drug buddies that also receive cocaine injections to more accurately reflect the social experience of addiction. Lastly, the quantification of animal behavior is very tedious and time-consuming work. If in the future this procedure becomes frequently used, methods of automated analysis should be explored as an addition to this social interaction chamber.

Although this is only the first step in formulating a model for social reinstatement, the knowledge that rats prefer to interact with conspecifics conditioned to be associated with cocaine is valuable to this development. An animal model designed to examine social reinstatement, and the neurobiological underpinnings of this phenomenon, has the potential to provide powerful insight to a relatively understudied contributing factor of drug relapse and to significantly advance the treatment of addiction and maintenance of abstinence.
